# Comprehensive analysis of m6A RNA methylation modification patterns and the immune microenvironment in osteoarthritis

**DOI:** 10.3389/fimmu.2023.1128459

**Published:** 2023-03-16

**Authors:** Zhixin Liu, Heng Liu, Deqiang Li, Liang Ma, Tongxin Lu, Hao Sun, Yuankai Zhang, Hui Yang

**Affiliations:** ^1^ Department of Orthopedics, Qilu Hospital of Shandong University, Jinan, Shandong, China; ^2^ NHC Key Laboratory of Otorhinolaryngology, Qilu Hospital of Shandong University, Jinan, Shandong, China; ^3^ Department of Otorhinolaryngology, Qilu Hospital of Shandong University, Jinan, Shandong, China; ^4^ Department of Radiology, Qilu Hospital of Shandong University, Jinan, Shandong, China

**Keywords:** osteoarthritis, m6A RNA methylation, epigenetics, immune microenvironment, bioinformatics

## Abstract

**Background:**

Osteoarthritis (OA) is the most common joint degenerative disease, and so far, there is no effective therapy to prevent or delay its development. Considerable attention is now being given to the impact of m6A RNA methylation modification on the disease immune regulation. However, much remains unknown about the function of m6A modification in OA.

**Methods:**

A total of 63 OA and 59 healthy samples were applied to comprehensively examine the m6A regulators mediated RNA methylation modification pattern in OA, and evaluate the impacts of distinct patterns on the characteristics of OA immune microenvironment, including immune infiltration cells, immune responses and human leukocyte antigen (HLAs) genes expression. In addition, we screened out the m6A phenotype-related genes and further explored their potential biological functions. At last, we verified the expression of key m6A regulators and their associations with immune cells, *in vitro*.

**Results:**

Most of m6A regulators was differentially expressed in OA samples compared to the normal tissues. Based on six hub-m6A regulators identified as abnormally expressed in OA samples, we developed a classifier to distinguish OA patients from healthy individuals. We noted that immune characteristics of OA were correlated with m6A regulators. For instance, YTHDF2 had a strongest significantly positive correlation with regulatory T cells (Tregs) and IGFBP2 was strongest negatively associated with dendritic cells (DCs), which were confirmed by the immunohistochemistry (IHC) staining. Two distinct m6A modification patterns were determined: pattern B had higher infiltrating immunocytes and more active immune responses than pattern A, and two patterns differed in the expression of HLA genes. We also identified 1,592 m6A phenotype-related genes that could mediate the OA synovitis and cartilage degradation by the PI3K-Akt signaling pathway. Quantitative real-time polymerase chain reaction (qRT-PCR) results indicated that IGFBP2 was significantly overexpressed, while YTHDF2 mRNA expression was decreased in OA samples, which was consistent with our findings.

**Conclusion:**

Our research proves the essential impact of m6A RNA methylation modification on the OA immune microenvironment, and helps to explain the regulatory mechanism behind it, which may open up a new direction for more precise immunotherapy of osteoarthritis.

## 1 Introduction

Osteoarthritis (OA) is the most prevalent joint degenerative disease, accompanied by pain, joint deformity and disability (1). It is the main cause of disability and reduction in life expectancy among the elderly (2, 3). Although joint replacement surgery is an option, there is as yet no effective therapy to prevent the OA progression (4). There are plenty of risk factors for its development, including genetic predisposition, aging, sex, obesity, trauma, anatomical abnormality, low-grade systemic inflammation and the failure of the oxidation-antioxidant balance (5–7). Among them, the low-grade chronic inflammation plays a central role. It has been reported that immune systems are involved in the low-grade inflammation related to OA, and the inflammation of OA tissues is the results of interaction between immune system, local tissue damage, metabolic dysfunction and other factors (8). Recent researches have demonstrated that the pathogenesis of OA has a strongly close correlation with the immune microenvironment, and the immune pathways as well as the infiltrating immunocytes in OA synovial tissue are linked with the pain and progression of OA (9). Woodell-May JE and Sommerfeld SD have revealed the huge potentials of T cells and macrophages in the synovial membrane to regulate the OA inflammatory response (10), and the relevant studies on the chondrocyte apoptosis and cartilage matrix proteolysis mediated by the immune responses and immune cells have also been reported (11). Further elucidating the immune regulation mechanism of OA may explain its potential pathogenesis and generate novel therapeutic methods that may modify its course.

RNA methylation is thought to be a key regulator of disease progression, and it has important impacts on many aspects of RNA metabolism, such as RNA splicing and subcellular localization, RNA degradation, mRNA stability and translation (12–14). N6-methyladenosine (m6A) modification is the most abundant internal modifications found in eukaryotes, which recognized by m6A binding proteins (readers), installed by methyltransferases (writers), and removed by demethylases (erasers) (15, 16). m6A RNA modification actively participates in several physiological processes, including DNA damage response, tumorigenesis, cell cycle progression, differentiation and circadian rhythm maintenance (17, 18). Recent studies have revealed that m6A RNA methylation shows a strong association with inflammation responses. Dubey et al. have demonstrated that the knockdown of m6A demethylase FTO contributes to hypermethylation of pro-inflammatory cytokines, and regulation of m6A−RNA methylation could effectively suppress myocardial inflammation and dysfunction during endotoxemia in mice (19). METTL3 is highly expressed in rheumatoid arthritis and leads to fibroblast-like synoviocytes (FLSs) activation and inflammatory response *via* the NF-κB signaling pathway (20). Inhibition of METTL3 obviously alleviates renal injury and inflammation through IGF2BP2-dependent mechanisms, which suggests that targeting METTL3 may be a promising therapeutic approach for acute kidney injury (AKI) (21). In hematopoietic stem cell (HSC), m6A reader YTHDF2 acts as a key repressor of proinflammatory pathways, and the crucial significance of m6A mRNA modification in mediating the inflammatory pathways of HSC has also been proved (22). Several m6A regulators such as ALKBH5, METTL3, and FTO have been reported to be involved in the progression of osteoarthritis and attenuate the inflammatory response, which provides a potential strategy for OA treatment (23–25). Evidences have shown that aberrant m6A RNA methylation modification is connected with the immune regulation, and m6A modification is the main regulator of post-transcriptional immune response in cells (26). m6A RNA methylation could mediate the tumor immunity by regulating the expression of antioncogenes and oncogenes, which may provide a highly targeted mechanism for tumor immunotherapy. Served as promising predictors, m6A RNA methylation regulators are able to identify pancreatic cancer (PAAD) patients who may really benefit from treatment with immunotherapy and enhance the efficacy of immunotherapy for PAAD, which would allow to refine the therapeutic options and to better adjust the treatment strategies (27). In addition, m6A RNA methylation modification pattern take part in the regulation of immune microenvironment of rheumatoid arthritis, suggesting it may have a potential role in the immunomodulation and immunotherapy for bone and joint diseases (28). However, there is still a gap in the study on the function of m6A in osteoarthritis, and the correlation of m6A modification with the OA immune microenvironment requires further elucidation.

Nowadays, the study on the epigenetic regulation mechanism of biological processes has contributed to the understanding of the pathogenesis of various human diseases caused by abnormal epi-transcriptomic and epigenomic modification. Specifically, the increasing findings have determined the connection between the pathogenesis of diseases and aberrant modifications. In our research, we comprehensively explored the m6A regulators mediated RNA methylation modification patterns in osteoarthritis, and determined the potential regulation mechanism of m6A RNA methylation modification on the OA immune microenvironment. Our findings have showed that m6A regulators could effectively differentiate OA from healthy samples and were closely related to infiltrating immunocytes and immune pathways in OA, indicating that m6A regulators could mediate the regulation of immune microenvironment in OA. Based on 18 m6A regulators, we clustered OA samples and screened out two m6A RNA methylation modification subtypes, with distinct biological functions and immune characteristics. Additionally, 1,592 m6A phenotype-related genes and their underlying biological functions as well as molecular mechanism were observed. *In vitro*, we verified the expression of key m6A regulators and their associations with immune cells. Our study suggests that m6A RNA methylation modification pattern is of vital importance in the immune microenvironment of OA, and this classification of immune subtypes based on m6A regulators may provide a theoretical basis for precise OA immunotherapy.

## 2 Materials and methods

### 2.1 Clinical samples

We collected 10 OA tissues from patients who undergoing total knee arthroplasty, and 10 normal samples were gained from patients with ligament injury who underwent ligament reconstruction surgery at Qilu Hospital of Shandong University, China. Patients were randomly selected and all signed informed consent. Our research was approved by the Ethics Committee of Qilu Hospital of Shandong University.

### 2.2 Data pre-processing

The datasets of gene expression profile were downloaded from the GEO database (https://www.ncbi.nlm.nih.gov/geo/): GSE82107, GSE29746, GSE55235, GSE55457, GSE117999 and GSE98918. A total of 63 OA samples and 59 healthy individuals were gained. The *sva* R package was applied to normalize the six gene sets (**Figure S1**). Detailed clinical features and platforms of patients could be found in **Supplementary Files 1.** 18 m6A regulators were annotated in the metadata sets: IGFBP2, YTHDF2, HNRNPA2B1, METTL3, YTHDC2, FTO, RBM15, FMR1, LRPPRC, IGFBP3, YTHDF1, HNRNPC, YTHDC1, ZC3H13, RBM15B, YTHDF3, IGFBP1 and WTAP.

### 2.3 Difference analysis of m6A RNA methylation regulators between OA and healthy patients

The protein-protein interaction (PPI) network among 18 m6A RNA methylation regulators was constructed using the STRING database. The correlations among m6A regulators expression in all samples or OA samples were determined by Spearman’s correlation. The *limma* R package was employed to screen out the differentially expressed genes (DEGs) between OA and normal tissues, and the cut-off criterion was P value < 0.05. Wilcox test was utilized to compare the differences in m6A regulators expression between OA and healthy donors. With a cut-off of P < 0.05, the univariate logistic regression was applied to find the m6A regulators linked with OA occurrence. Feature selection and dimension reduction were performed by the least absolute shrinkage and selection operator (LASSO) Cox regression (10-fold). Subsequently, the results of the univariate logistic and LASSO regression overlapped, and the corresponding coefficients of m6A RNA methylation regulators were computed using multivariate logistic regression. The performance of risk score in distinguishing OA from healthy patients was evaluated by ROC curve analysis *via* the *pROC* package in R. Values of the area under the curve (AUC) > 0.7 and P < 0.05 suggested that it had high predictive ability.

### 2.4 Correlation analysis of m6A RNA methylation regulators with immune characteristics in OA

The reactivity and number of immune infiltration cells as well as the activity of the immune responses were detected using the single-sample gene-set enrichment analysis (GSEA), *via* the *gsva* package in R. Wilcox tests was applied to compare the infiltrating immunocytes abundance and the enrichment fraction of immune response pathways between OA and normal patients. Using the *corrplot R* package, connections between hub m6A regulators, infiltrating immunocytes and immune response pathways were examined by Spearman correlation test.

### 2.5 Identification of m6A RNA methylation modification patterns in OA

The unsupervised consensus cluster analysis was implemented to identify the m6A modification pattern in OA samples, based on the 18 m6A RNA methylation regulators. A consensus clustering algorithm was employed to assess the number and robustness of cluster, and the *consul cluster plus* package in R for 1,000 iterations to ensure classification robustness. The expression pattern of 18 m6A RNA methylation regulators in different m6A modification pattern was confirmed *via* the principal component analysis (PCA). Next, 18 m6A regulators expression, infiltrating immunocytes abundance, immune responses and HLA genes expression of the two different m6A subtypes were compared by the Kruskal test.

### 2.6 Functional enrichment analysis of distinct m6A RNA methylation modification patterns

The biological function of distinct m6A RNA methylation modification pattern was analyzed *via* the *clusterProfiler* R package, including GO, KEGG and Hallmark enrichment analysis. The c5.go.v7.4.symbols, c2.cp.kegg.v7.4.symbols and h.all.v7.4.symbols gene sets were employed to reflect alterations of the biological signaling pathways. The GSVA algorithm was applied to convert the expression matrix into a scoring matrix, and the *limma* package in R was utlized to compare the scores of biological signaling pathways among these groups. P < 0.05 was selected as the threshold of difference analysis.

### 2.7 Identification of genes mediated by m6A RNA methylation regulators in OA

All samples in different m6A RNA methylation modification pattern was determined using the empirical Bayesian method to screen out DEGs between the two m6A subtypes of OA. The adjusted P < 0.0001 was applied to find the significant DEGs. m6A modification pattern-related genes and gene modules were constructed by weighted gene co-expression network analysis (WGCNA). The topology was calculated with a soft threshold of 1 to 20, and 10 was set as the optimal soft threshold. According to the topological overlap matrix (TOM), the related modules were classified. Each module contains at least 20 genes, and then similar modules were combined. The correlation between the combined modules and distinct m6A modification patterns was computed using the Pearson approach.

### 2.8 Immunohistochemistry staining

The correlations between the expression of YTHDF2 and Treg marker FOXP3, IGFBP2 and DCs marker CD1a were verified by IHC staining. The sections of OA and normal tissues were added into 10 mM sodium citrate solution (pH 6.0) for deparaffinization and antigen retrieval, and blocked with PBS containing 5% goat serum for 30 min RT. The primary antibody diluted in 1:200 was added into the slides and incubated overnight at 4°C, followed by incubation with the HRP-conjugated secondary antibody and stained with the 3.30-diaminobenzidine (DAB) substrate. The tissue score (IHC score) was calculated based on the percentage of stained cells and tissue staining intensity, and the intensity scoring standards were as follows: strong (+++), moderate (++), weak (+), and negative (–). YTHDF2 polyclonal antibody (24744-1-AP, Proteintech Group, Inc.) was used to detect YTHDF2. IGFBP2 polyclonal antibody (11065-3-AP, Proteintech Group, Inc.) was utilized to detect IGFBP2. FOXP3 monoclonal antibody (YM0286, ImmunoWay Biotechnology, Inc.) was applied to examine Treg marker FOXP3. CD1 monoclonal antibody (YM3068, ImmunoWay Biotechnology, Inc.) was employed to examine DCs marker CD1a. Mouse IgG (Santa Cruz, sc-2025) and rabbit IgG (Proteintech, B900610) were used as negative controls. CoraLite488 conjugated Affinipure goat anti-mouse IgG (H+L) (SA00013-1, Proteintech Group, Inc.) and CoraLite594 conjugated goat anti-rabbit IgG (H+L) (SA00013-4, Proteintech Group, Inc.) were used as secondary antibodies.

### 2.9 RNA extraction and qRT-PCR

Total RNAs was gained by applying 1 ml TRIzol reagent (Sigma-Aldrich) from OA and normal tissues, then reverse transcribed into cDNA using a reverse transcription kit (TaKaRa). IGFBP2 and YTHDF2 mRNA expression were detected by qRT-PCR with SYBR Green PCR Master Mixes (Thermo Fisher). The 2−ΔΔCt method was applied to calculate the relative quantification and select β-Actin as the internal control. The sequences of YTHDF2 were as follows: AGCCTCTTGGAGCAGTACAAA (sense) and GCATTATTGGGCCTTGCCTG (antisense). The sequences of IGFBP2 were as follows: GAGCAGGTTGCAGACAATGGC (sense) and TACCCGACTTGAGGGGCTT (antisense). The sequences of ß-actin were as follows: CACCATTGGCAATGAGCGGTTC (sense) and AGGTCTTTGCGGATGTCCACGT (antisense).

## 3 Results

### 3.1 Expression landscape of m6A RNA methylation regulators in OA and healthy samples

**Figure 1:**
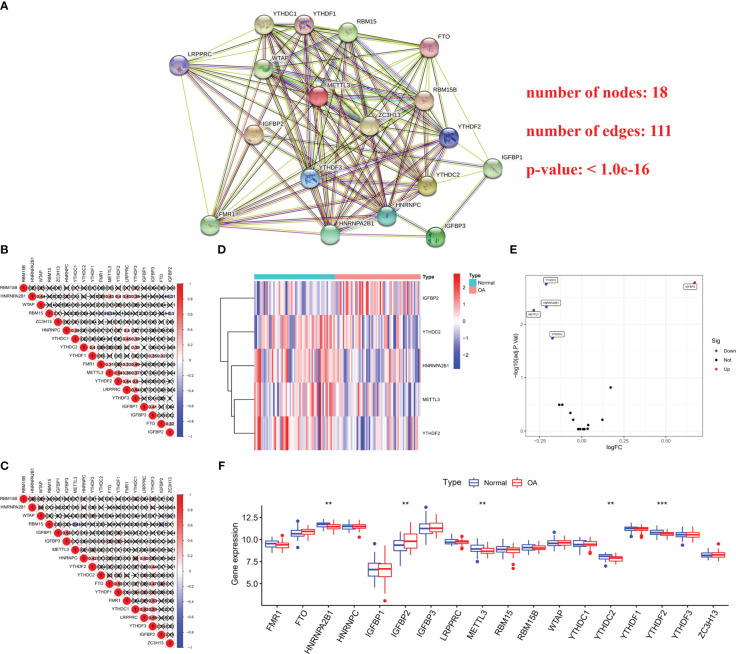
Expression landscape of m6A regulators in OA and healthy samples. **(A)** The protein-protein interaction (PPI) network of 18 m6A regulators. **(B)** Connection analysis between 18 m6A regulators expression in all samples. **(C)** Connection analysis between 18 m6A regulators expression in OA samples. **(D, E)** Heat map and volcano map of differences in hub m6A regulators expression between health and OA patients. **(F)** Box plot showing 18 m6A regulators expression in OA samples compared with the control. **p < 0.01, and ***p < 0.001.

A total of 18 m6A regulators were screened out, including five writers (WTAP, ZC3H13, RBM15B, RBM15 and METTL3), 12 readers (FMR1, LRPPRC, YTHDC1, YTHDC2, HNRNPA2B1, HNRNPC, YTHDF1, YTHDF2, YTHDF3, IGFBP1, IGFBP2 and IGFBP3) and one eraser (FTO). The protein-protein interaction (PPI) network described the close connections among 18 m6A regulators, which suggested that the cross-talk among them may affect the formation of m6A modification pattern and participate in the occurrence and development of OA (**Figure 1A**). Next, we studied the relationship between transcriptomes by computing the pairwise relation between 18 m6A regulators expression, and noticed that positive connections were more frequent than negative ones, and the strongest positive correlation in all samples was between YTHDF3 and LRPPRC (r = 0.54, **Figure 1B**). In the OA samples, YTHDF3 and LRPPRC also showed the strongest positive correlation (r = 0.59, **Figure 1C**), indicating their important role in the m6A RNA methylation. The differential expression analysis revealed five m6A regulators (YTHDF2, HNRNFA2B1, METTL3, YTHDC2 and IGFBP2) with altered expressions in OA samples, compared with the healthy control (**Figure 1D**). Amongst these, the multiple change in IGFBP2 was the largest (**Figure 1E**). In addition, Wilcox test showed the significant difference in m6A regulators expression between OA and normal tissues, including YTHDF2, HNRNFA2B1, METTL3, YTHDC2 and IGFBP2 (**Figure 1F**). The above findings demonstrated that m6A RNA methylation regulators expression was highly heterogeneous in OA patients and healthy individuals, suggesting the potential functions of the abnormal m6A regulators expression patterns in the progression of OA.

### 3.2 m6A RNA methylation regulators were involved in the OA progression

**Figure 2:**
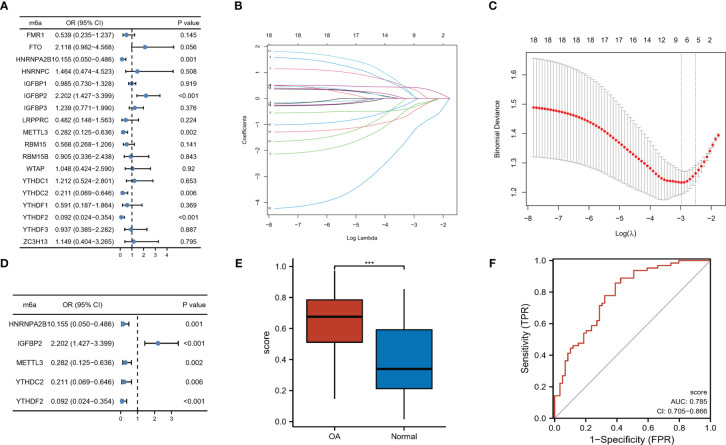
m6A regulators were linked with the OA process. **(A)** Five m6A regulators showed close correlations with the occurrence of OA through univariate logistic regression (P < 0.05). **(B)** LASSO coefficient profiles of the five OA-related m6A regulators. **(C)** In LASSO regression 10-fold cross-validation applied to turn parameter selection. **(D)** Multivariate logistic regression to establish the distinguishing signatures of five m6A regulators and calculate risk scores for OA. **(E)** Risk profiles of OA and healthy samples. **(F)** The ROC curve analysis of hub m6A regulators for OA diagnosis. ***p < 0.001.

To determine the function of m6A RNA methylation regulators in the pathogenesis of OA, the univariate logistic regression analysis of 18 m6A regulators was carried out. This suggested that the above five expressed differentially m6A regulators were related most closely to OA occurrence (P < 0.05, **Figure 2A**). LASSO regression was utilized for feature selection and dimension reduction to eliminate redundant genes (**Figures 2B, C**), and the results indicated these five regulators were all important for OA. We then developed a classifier composed of five m6A modulators that are able to distinguish OA samples from healthy samples using the multivariate logistic regression based on the risk scores, where the risk scores of OA samples are much higher than those of healthy group (**Figures 2D, E**). Risk scores were calculated as expression of HNRNPA2B1*-0.5548 + expression of IGFBP2*0.8090 + expression of METTL3*-0.5232 + expression of YTHDC2*-0.8723 + expression of YTHDF2*-1.7990. Analysis of ROC curve suggested that this classifier showed a good predictive performance on the diagnosis of osteoarthritis (AUC = 0.785, **Figure 2F**). We concluded that m6A RNA methylation regulators could be identified as potential indicators and participated in the process of OA.

### 3.3 Correlation analysis between m6A regulators and immune characteristics in OA

**Figure 3:**
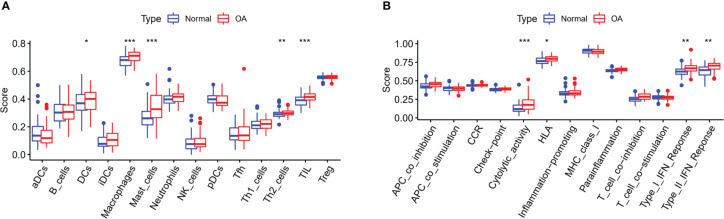
Differential expressions of immunocytes and immune-related response pathways in OA. **(A)** Differential expressions of immunocytes in OA compared to the healthy samples. **(B)** Differential expressions of immune response pathways in OA compared to the healthy samples. *p < 0.05, **p < 0.01, and ***p < 0.001.

**Figure 4:**
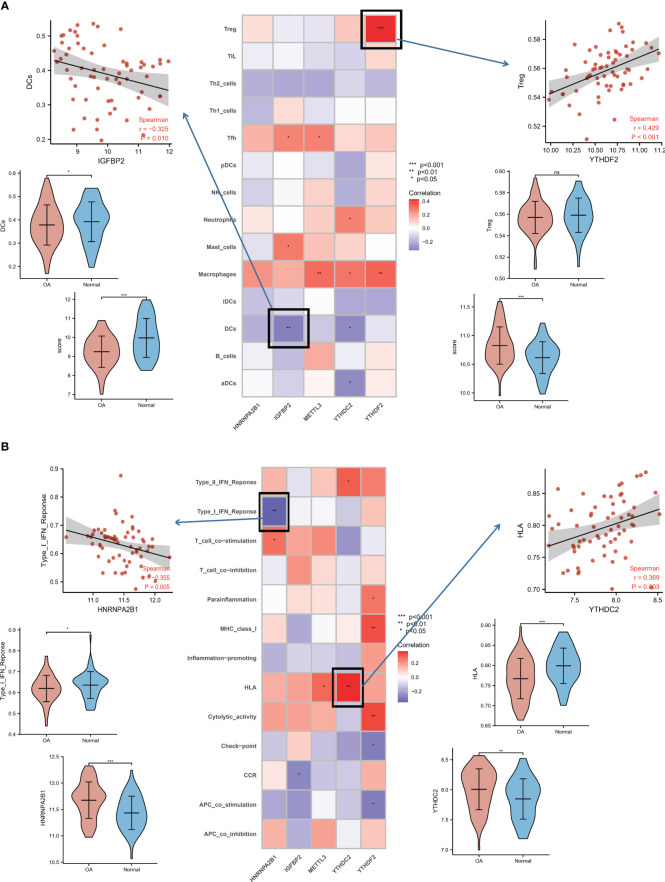
Relationship analysis of m6A regulators with immune characteristics in OA. **(A)** Dot plot showing the relationship of infiltrating immune cells with hub m6A regulators in the OA immune microenvironment. The most positively related immunocyte-m6A regulator pair is YTHDF2-Treg cell (r = 0.429), and the most negative is IGFBP2-DC cell (r = -0.325), with expression and fraction status presented in the upper panel. **(B)** Dot plot showing the relationship of immune response pathways with hub m6A regulators in the OA immune microenvironment. The most positively associated pair is the YTHDF2-HLA pathway (r = 0.369), the most negatively correlated is HNRNPA2B1-Type I IFN response (r = -0.355), with the violin plot in the bottom panel showing expression or activity.

**Figure 5:**
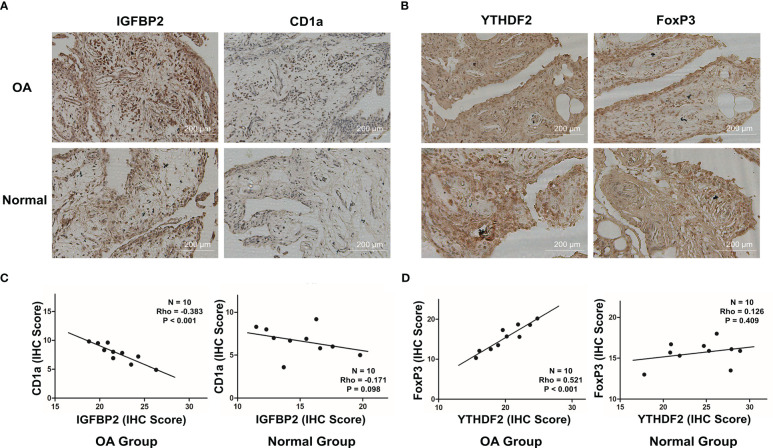
Correlations between the expression of IGFBP2 and DCs marker CD1a, YTHDF2 and Treg marker FOXP3 in OA and normal tissues. **(A)** Representative images for the IHC staining of IGFBP2 and CD1a in OA and normal tissues (n = 10). **(B)** Representative images for the IHC staining of YTHDF2 and FOXP3 in OA and normal tissues (n = 10). **(C)** Relation between protein levels of IGFBP2 and CD1a in OA and normal tissues *via* Spearman correlation. **(D)** Relation between protein levels of YTHDF2 and FOXP3 in OA and normal tissues *via* Spearman correlation.

The connections between dysregulated m6A RNA methylation regulators and immune infiltration cells as well as immune response pathways were analyzed, to study its contribution to the OA immune microenvironment. Difference analysis showed that the abundance of most infiltrating immunocytes in OA samples was significantly higher than that in health, such as dendritic cells (DCs), macrophages, mast cells, TIL and Th2 cells (**Figure 3A**). And we found the five hub m6A regulators had close connections to several immune cells in OA samples using the correlation analysis (**Figure 4A**). For example, YTHDF2 showed the strongest positive relation to regulatory T cells (Tregs, r = 0.429), and the strongest negative correlation was between IGFBP2 and DCs (r = -0.325). Subsequently, we collected 10 OA samples and 10 normal tissues, and the results of IHC staining indicated that the expression of IGFBP2 in OA tissues was higher than that of the normal, while YTHDF2 expression was decreased in OA group. In OA tissues, correlation analysis suggested that IGFBP2 was negatively linked to DCs marker CD1a (**Figures 5A, C**), and the positive correlation between YTHDF2 and Treg marker FOXP3 was also confirmed (**Figures 5B, D**). The immune response of OA tissues was more active than that of healthy samples, including HLA, Cytolytic activity, Type I and Type Π IFN inflammatory responses, which suggest that these pathways might participate in the inflammatory process of OA (**Figure 3B**). We further analyzed the differences in other inflammatory response pathways between OA and normal samples (**Figure S2**). In addition, YTHDF2 had the strongest positive relation to the HLA pathway (r = 0.369), HNRNPA2B1 and Type I IFN response showed the strongest negative correlation (r = -0.355, **Figure 4B**). These results indicated that m6A RNA methylation regulators were of essential importance in the immune microenvironment of OA and could mediate the infiltration of immune cells and the responses to immunity in OA.

### 3.4 Identification of m6A RNA methylation modification patterns

**Figure 6:**
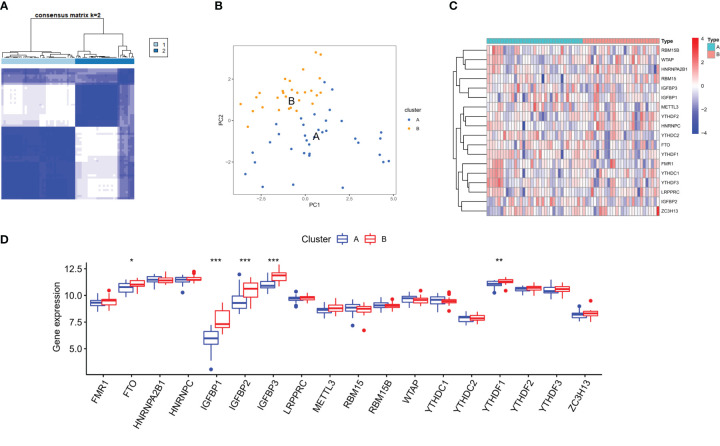
Distinct m6A RNA methylation modification pattern of OA. **(A)** Two different m6A subtypes of OA by the unsupervised consensus cluster analysis. **(B)** Principal component analysis of the transcriptome profiles indicated the two subtypes were significantly different. **(C, D)** Heatmap and box plot of 18 m6A regulators expression between the two m6A modification subtypes of OA. *p < 0.05, **p < 0.01, and ***p < 0.001.

**Figure 7:**
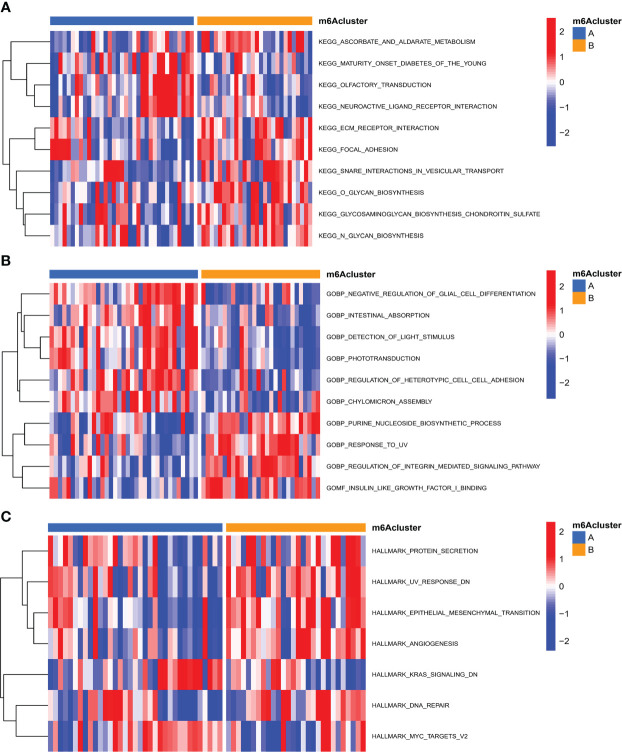
Functional analysis of different m6A RNA methylation modification patterns in OA. **(A)** Differences in KEGG pathway enrichment scores. **(B)** Differences in GO pathway enrichment scores. **(C)** Differences in Hallmark analysis scores.

On the basis of 18 m6A regulators expression, the unsupervised consensus cluster analysis was carried out to detect m6A RNA methylation modification patterns in OA patients, and two different subtypes were found in our study (**Figure 6A**). PCA analysis showed that m6A regulators expression was qualitatively different, and m6A regulators effectively enabled classification of OA patients into two groups, with 35 samples in subtype A and 28 samples in subtype B (**Figure 6B**). Most of m6A regulators expression was significantly different between two m6A subtypes of OA, with FTO, IGFBP2, IGFBP1, IGFBP3 and YTHDF1 being highly expressed in subtype B (**Figures 6C, D**). We further investigated the underlying biological processes and molecular mechanisms in the different m6A RNA methylation modification pattern of OA, and KEGG pathway analysis suggested that pattern B showed more active pathways than pattern A, such as FOCAL ADHESION and ECM RECEPTOR INTERACTION (**Figure 7A**). And GO analysis indicated that pattern B also had more abundant biological processes, compared to the pattern A, including PURINE NUCLEOSIDE BIOSYNTHETIC PROCESS and REGULATION OF INTERGRIN MEDIATED SIGNALING PATHWAY (**Figure 7B**). In the Hallmark analysis, PROTEIN SECRETION and EPITHELIAL MESENCHYMAL TRANSITION were enriched in pattern B (**Figure 7C**). These findings have revealed the presence of diverse m6A modification patterns in OA, which performed different biological functions in the progression of OA.

### 3.5 Characteristic of the immune microenvironment in distinct m6A modification pattern

**Figure 8:**
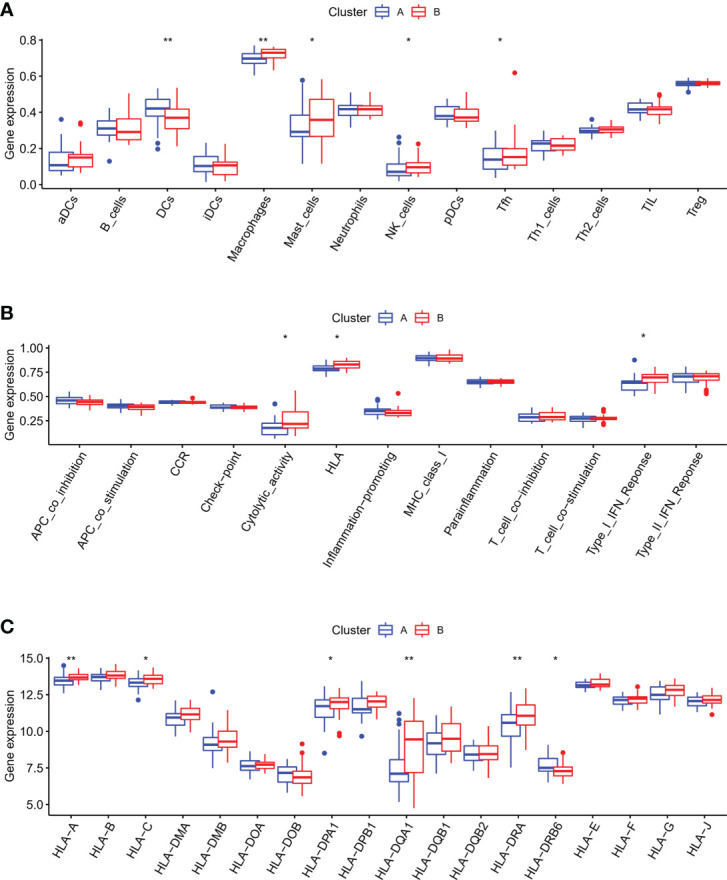
Characteristics of the immune microenvironment in two different m6A subtypes of OA. **(A)** Differences in the infiltrating immunocytes in the immune microenvironment of distinct m6A subtypes in OA. **(B)** Differences in the immune pathways in the immune microenvironment of distinct m6A subtypes in OA. **(C)** Differences in the HLA genes expression in the immune microenvironment of distinct m6A subtypes in OA. *p < 0.05, and **p < 0.01.

Next, we explored the differences in the characteristics of the immune microenvironment between two m6A modification patterns in OA, and examined the differences in the infiltrating immunocytes abundance immune response pathways and HLA genes expression. Our results demonstrated that pattern B had a relatively high infiltrating immunocytes compared with pattern A, and higher infiltration levels of macrophages, NK cells, mast cells and Tfh, whereas DCs were mainly involved in the pattern A (**Figure 8A**). The immune response of pattern B was also more active than that of pattern A, with regard to Cytolytic activity, HLA and Type I IFN response (**Figure 8B**). HLA genes expression was significantly different in two different m6A modification subtypes of OA (**Figure 8C**). We concluded that different m6A modification pattern mediated immune response in OA was distinguishing, pattern B mediated an active immune response while pattern A led to a mild in the OA immune microenvironment. It was verified that m6A RNA methylation modification played an essential regulatory role in the formation of different immune microenvironments of OA.

### 3.6 Identification of m6A phenotype-related genes in OA

**Figure 9:**
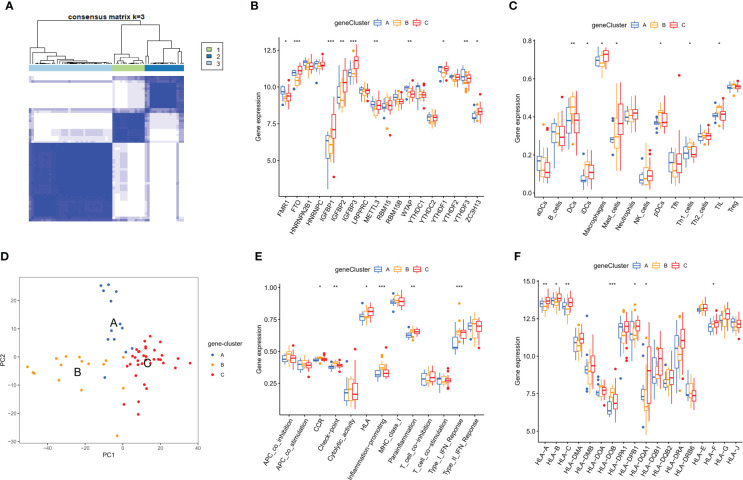
Characteristics of the immune microenvironment in m6A phenotype-related genes modification patterns of OA. **(A)** Three different m6A phenotype-related genes modification subtypes of OA by the unsupervised consensus cluster analysis. **(B)** Principal component analysis of the transcriptome profiles indicated the three subtypes were significantly different. **(C-F)** Differential expressions of 18 m6A regulators, immunocytes, immune response pathways and HLA genes in the immune microenvironment of m6A phenotype-related genes modification patterns in OA. *p < 0.05, **p < 0.01, and ***p < 0.001.

To determine the potential mechanism of genes involvement in the regulation mediated by m6A regulators, we screened out m6A phenotype-related genes, amounting to a total of 1,592 genes. On the basis of the expressions of these genes, we performed the unsupervised consensus cluster analysis in OA samples, and three gene subtypes were identified (**Figure 9A**). PCA analysis showed three groups of OA patients, with 13 samples in subtype A, 16 in subtype B and 34 in subtype C (**Figure 9B**). We noticed the significant differences between three subtypes in 18 m6A regulators expression, immune infiltrating cells, immune pathways and HLA genes expression. Most of m6A regulators was expressed differentially among the three subtypes (**Figure 9C**). Compared to the patterns B and C, pattern A showed the fewest abundance of infiltrated immune cells. Pattern B had higher infiltrated levels of DCs, iDCs, pDCs, TIL and Th1 cells, while macrophages and mast cells were enriched in pattern C (**Figure 9D**). Patterns B and C had more active immune reactions than pattern A, especially pattern B. CCR, Check-point, Inflammation-promoting, Para-inflammation and Type I IFN response were very active in pattern B, as was HLA in pattern C (**Figure 9E**). Moreover, except for HLA-DOB, the expression of HLA genes was higher in pattern C than in pattern A and pattern B (**Figure 9F**).

**Figure 10:**
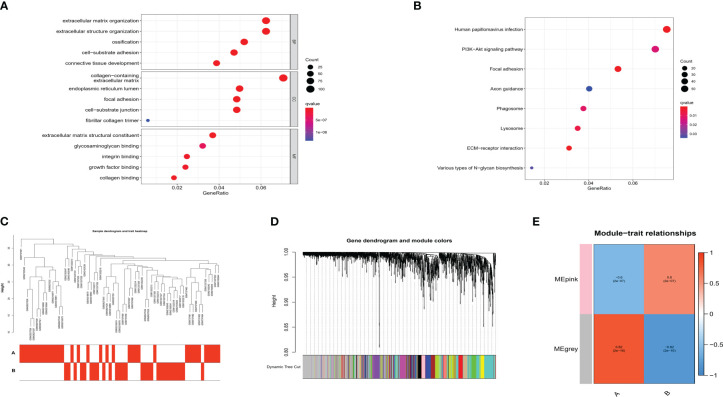
Functional analysis of m6A phenotype-related genes in OA. **(A)** GO enrichment analysis. **(B)** KEGG enrichment analysis. **(C)** Sample dendrogram and trait heatmap using WGCNA. **(D)** Hierarchical clustering tree shows each module. **(E)** Heatmap shows the relationship of m6A RNA methylation modification pattern with module eigengenes.

We then performed functional enrichment analysis on the m6A phenotype-related genes, and the results of GO analysis showed them to be mainly involved in the extracellular matrix organization (**Figure 10A**). In the KEGG analysis, these genes were mostly enriched in the PI3K-Akt signaling pathway (**Figure 10B**). Using WGCNA to screen out gene-gene modules related to the distinct m6A modifications in OA (**Figures 10C, D**), and we identified two gene modules in which different m6A modification patterns were matched with their related genes (**Figure 10E**). These findings may shed light on the regulatory network of gene expression mediated by m6A modification patterns.

### 3.7 qRT-PCR validation of key m6A regulators

**Figure 11:**
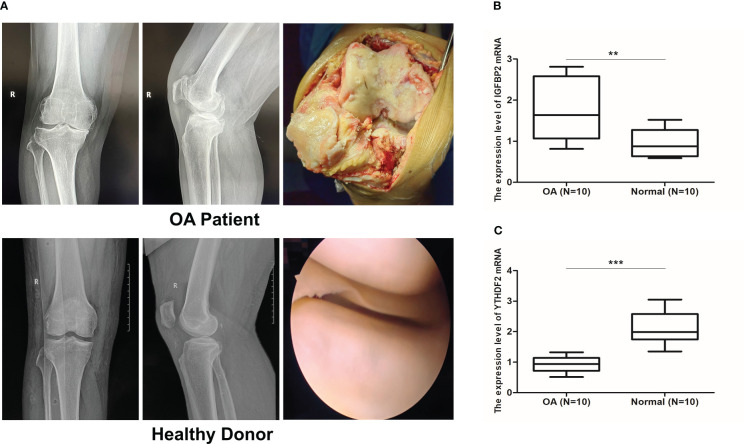
qRT-PCR validation of key m6A regulators. **(A)** X ray images and intraoperative macroscopic views (arthroplasty and arthroscopy image) of knee joint from OA patients and healthy donors. **(B, C)** Validation of IGFBP2 and YTHDF2 mRNA expression by qRT-PCR between OA (n = 10) and normal group (n = 10). **p < 0.01, and ***p < 0.001.

Radiological evaluations of knee joints were performed in distinct groups of patients to validate the typical imaging manifestations of OA. Our study noticed that preoperative knee X-ray and intraoperative macroscopic images of OA patients showed joint space stenosis, cartilage degeneration, subcartilage bone hyperplasia, and osteophyte formation in comparison to those of the healthy donors. (**Figure 11A**). The results of qRT-PCR showed that compared the normal group, the expression of IGFBP2 mRNA was significantly up-regulated, while YTHDF2 mRNA expression was decreased in OA samples, which was consistent with our findings (P < 0.01, **Figures 11B, C**).

## 4 Discussion

Osteoarthritis (OA) is considered as a degenerative inflammatory disease of the joint cartilage, with inflammatory mediators released by synovium, bone and cartilage (29). The inflammatory response of OA has been implicated in its pathogenesis, and elaborating on the inflammatory pathophysiology of osteoarthritis may represent new avenues for OA therapy (30). Extensive studies indicated that immunocytes and immune pathways effectively modulated the inflammatory response of OA and correlated with OA disease pain and progression (9). One of the m6A regulators, demethylase METTL3 could contribute to the development of OA by mediating the inflammatory response, ECM synthesis and chondrocyte apoptosis, which suggested that targeting of METTL3 or m6A RNA methylation may be a promising preventive and therapeutic approach for OA (31). In addition, m6A RNA methylation modification reportedly was involved in the immune responses of numerous diseases (32), and its functions in the immune microenvironment of various tumors had also been proved (33–35). Thus, we had reason to believe that m6A modification was connected with the regulation of OA immune microenvironment. We combined a variety of bioinformatics methods and experiments to study the m6A modification pattern in the OA immune microenvironment, and determined how m6A regulators mediated the abundance of immunocytes and immune responses in OA.

PPI networks showed that 18 m6A regulators significantly interacted with each other, suggesting that readers, erasers and writers do function in collaboration, in osteoarthritis, not in isolation. Correlation analysis has revealed that the strongest positive correlation among 18 m6A regulators was YTHDF3 and LRPPRC in both OA and all samples, which reminded us their important role in the m6A RNA methylation. Compared to the healthy samples, we identified five m6A regulators with altered expressions in OA patients, suggesting that these regulators may be related to the occurrence and development of OA. Based on the hub OA-related m6A regulators, we have created a novel classifier that could effectively distinguish OA from normal samples. These results further verified the crucial function of m6A regulators in osteoarthritis, and it could be identified as an independent diagnostic indicator of OA. We conducted qRT-PCR to validate the expression of key m6A regulators and the results proved that compared with the normal tissues, IGFBP2 mRNA was highly expressed while the mRNA expression of YTHDF2 was significantly down-regulated in OA patients, which was consistent with our findings. Subsequently, our study examined the differences in the characteristics of immune microenvironment between OA and healthy patients, including infiltrating immunocytes and immune response, and explored the potential relation of hub m6A regulators with immune characteristics in OA. We noted that the abundance of immune infiltration cells in OA tissues was higher than that in healthy individuals, and the immune response of OA group was also more active. In addition, we found YTHDF2 had the strongest positive connection with Treg cells, and IGFBP2 was strongest negatively associated with DCs. We collected samples from OA patients, and performed the IHC staining to confirm these correlations. The results showed that in OA tissues, YTHDF2 was positively linked with Treg marker FOXP3, and the negative correlation between IGFBP2 and DCs marker CD1a was also proved. Shao et al. have shown that YTHDF2 expression was positively associated with Treg markers FOXP3, CCR8, TGFB1 and STAT5B, and the activation of Treg cells by YTHDF2 led to a poor prognosis of liver hepatocellular carcinoma (36). IGFBP2 significantly facilitated the invasion and metastasis of pancreatic ductal adenocarcinoma *via* the NF-κB signaling pathway (37). IGFBP2 was correlated with M2 Macrophages, CD8+ T cells, which enhanced the immunosuppression of glioblastoma and it might be an effective immunotherapeutic strategy for glioblastoma therapy (38); However, there are no similar reports relating to IGFBP2 and DCs. Further studies were required to examine the potential mechanism of m6A regulators and Tregs as well as DCs in the immune regulation of OA. In terms of immune response, YTHDF2 was strongest positively connected with the HLA pathway, and HNRNPA2B1 was strongest negatively linked to the Type I IFN response, indicating the involvement of YTHDF2 and HNRNPA2B1 in the OA progression. The above findings revealed that the cross-talk among m6A regulators had the crucial function in the occurrence of OA, and might influence on regulating the immune microenvironment of OA.

On the base of 18 m6A regulators expression, the unsupervised consensus cluster analysis was carried out and two m6A modification patterns with different immune phenotypes in OA were identified. In the pattern B, the infiltration abundance of DCs, Tfh, NK cells, macrophages and mast cells were higher than that of pattern A. The immune response of pattern B was also more active, compared with the pattern A, which activated in the HLA and Type I IFN signaling pathways, reported in many chronic inflammatory diseases. And HLA genes expression significantly differed between the two m6A subtypes of OA. This classification of immune subtypes has provided insights into the regulation mechanism of the immune microenvironment in OA, which may be conducive to the formulation of more appropriate immunotherapy strategies for OA. This was similar to the research classifying gastric cancer into three m6A RNA methylation modification subtypes, which contributed to understand the regulation mechanism of tumor microenvironment and made more effective therapeutic methods for gastric cancer (39). In the periodontitis, different m6A RNA methylation modification pattern was linked with the phenotypic features and had a profound influence on the complexity and heterogeneity of the periodontitis immune microenvironment (40). We compared the biological functions of the two different m6A subtypes of OA, and enrichment analysis showed that pattern B had more biological processes and abundant pathways than pattern A, including ECM receptor interaction, purine nucleoside biosynthetic process and protein secretion. The above findings could clarify how m6A modification patterns mediated the regulation network of gene expression, which may elucidate the OA pathogenesis from m6A RNA methylation modification point of view. Subsequently, we identified 1,592 m6A phenotype-related genes that were mostly enriched in the PI3K-Akt signaling pathway. Recently studies have shown that the PI3K/Akt signaling pathway had a vital function in the subchondral bone dysfunction, cartilage degradation and synovial inflammation of OA, and affected the development of OA (41, 42). Xue et al. suggested that blocking the PI3K-AKT-mTOR pathway could effectively alleviate inflammatory reaction and induce chondrocytes to autophagy in OA (43). Having divided these genes into three m6A phenotype-related genes modification patterns, we realized the significant differences in m6A regulators expression, immune infiltrating cells, immune responses and HLA genes expression among these subtypes. By WGCNA analysis, we screened out the gene-gene modules linked with the distinct m6A RNA methylation modifications, and noticed that pattern A was closely associated with the grey module, indicating that the gene expression in this module was greatly influenced by m6A RNA methylation regulators. These results unraveled the regulatory network of gene expression mediated by m6A modification patterns, and contributed to understanding the pathologic mechanisms of OA.

We comprehensively examined the potential relation of m6A modification pattern with the OA immune microenvironment, confirming that m6A RNA methylation modification was closely connected with the OA process and had a regulatory impact on the immune microenvironment of OA. Our study filled the gap in the field of OA epigenetics, and raised a novel direction for the study about m6A modification of the immune-related pathogenesis in OA. Our studies of the genes associated with m6A modification patterns deepened our comprehension of the functional mechanism of m6A RNA methylation modification in OA, which may offer clues to appropriate therapeutic interventions and play critical functions in alleviating or treating OA. Nevertheless, our research had some limitations. The size of samples was limited, so our research results required verification through further experiments. All the data was from the mRNA expression values of genes and not directly reflected their protein expression levels, which might lead to ineffective evaluation of the immune response pathways based on protein expression. Although such limitations should not be ignored, our study did confirm the significant influence of m6A RNA methylation modification on the OA immune microenvironment and helped explain the underlying pathological mechanism.

## 5 Conclusions

In conclusion, our study found that m6A RNA methylation regulators showed a strong influence on the immune characteristics of OA, and uncovered the prospective regulatory mechanism of m6A modification in the OA immune microenvironment, which helped to better understand the pathological mechanism of OA, with implications for more precise immunotherapy of OA in the future.

## Data availability statement

The datasets presented in this study can be found in online repositories. The names of the repository/repositories and accession number(s) can be found in the article/**Supplementary Material**.

## Ethics statement

The studies involving human participants were reviewed and approved by the Ethics Committee of Qilu Hospital of Shandong University. The patients/participants provided their written informed consent to participate in this study. Written informed consent was obtained from the individual(s) for the publication of any potentially identifiable images or data included in this article.

## Author contributions

HY and YZ designed this study and reviewed the manuscript. ZL and HL contributed to bioinformatics analysis. DL and LM collected clinical samples and did statistical analysis. TL and HS prepared the manuscript and performed the experiments. All authors contributed to the article and approved the submitted version.

## Supplementary material

Supplementary Materials 1 Datasets of GSE82107, GSE29746, GSE55235, GSE55457, GSE117999 and GSE98918 downloaded from GEO database.

Supplementary Figure 1 The datasets were combined, then normalized the original data by the *sva* package in R.

Supplementary Figure 2 Differences in other inflammatory response pathways between OA and normal samples.
