# Supplementary figures and images for: Comprehensive analysis of m6A RNA methylation modification patterns and the immune microenvironment in osteoarthritis
Source: Front Immunol. 2023 Mar 16;14:1128459. doi: 10.3389/fimmu.2023.1128459 (PMC10062708; doi:10.3389/fimmu.2023.1128459)

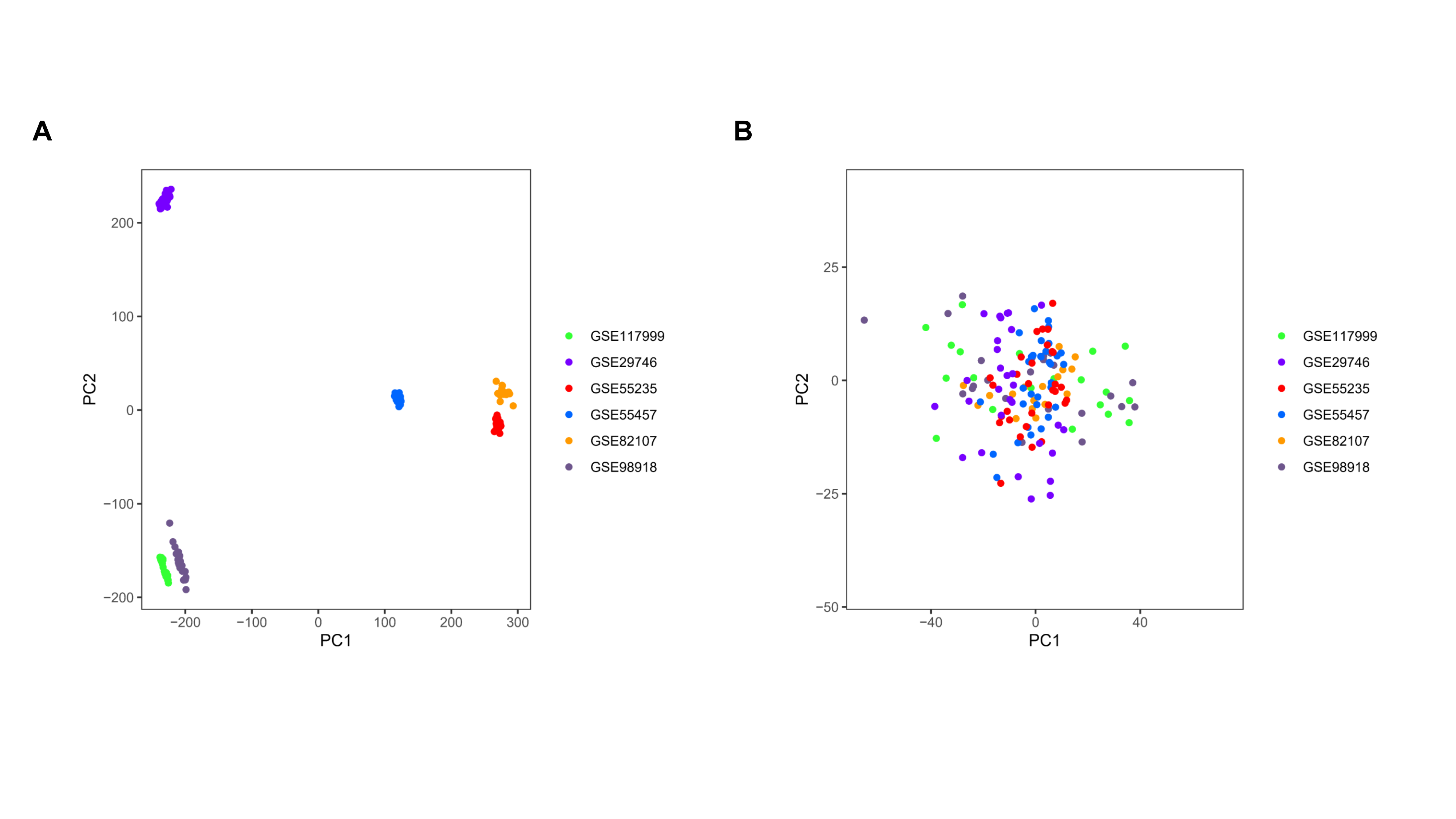

Supplement: Supplementary Figure 1 — The datasets were combined, then normalized the original data by the sva package in R. [file Image_1.tiff]

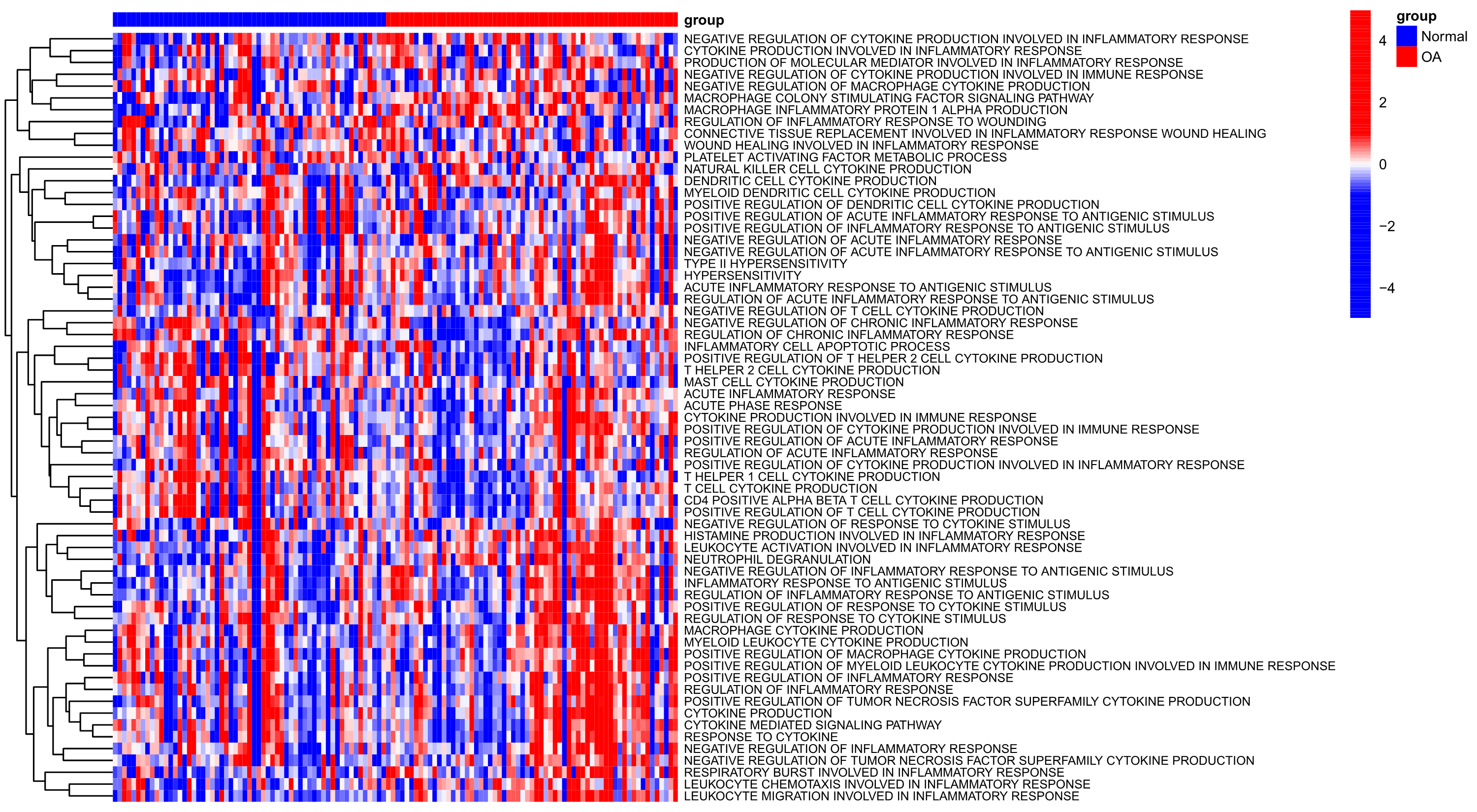

Supplement: Supplementary Figure 2 — Differences in other inflammatory response pathways between OA and normal samples. [file Image_2.tif]
